# Hepatocellular carcinoma-derived exosomal miRNA-21 contributes to tumor progression by converting hepatocyte stellate cells to cancer-associated fibroblasts

**DOI:** 10.1186/s13046-018-0965-2

**Published:** 2018-12-27

**Authors:** Yuan Zhou, Haozhen Ren, Bo Dai, Jun Li, Longcheng Shang, Jianfei Huang, Xiaolei Shi

**Affiliations:** 1 Department of Hepatobiliary Surgery, Affiliated Drum Tower Hospital of Nanjing University Medical School, 321, Zhongshan Road, Nanjing, 210008 Jiangsu Province China; 2 Department of Clinical Biobank, Nantong University Affiliated Hospital, 20, Xisi Road, Nantong, 226001 Jiangsu Province China

**Keywords:** Hepatocellular carcinoma, Hepatic stellate cells, Cancer associated fibroblasts, Exosome, miRNA-21, PTEN, AKT, Angiogenesis

## Abstract

**Background:**

Hepatocellular carcinoma (HCC) remains a global challenge due to its high morbidity and mortality rates as well as poor response to treatment. The communication between tumor-derived elements and stroma plays a critical role in facilitating cancer progression of HCC. Exosomes are small extracellular vesicles (EVs) that are released from the cells upon fusion of multivesicular bodies with the plasma membrane. There is emerging evidence indicating that exosomes play a central role in cell-to-cell communication. Much attention has been paid to exosomes since they are found to transport bioactive proteins, messenger RNA (mRNAs) and microRNA (miRNAs) that can be transferred in active form to adjacent cells or to distant organs. However, the mechanisms underlying such cancer progression remain largely unexplored.

**Methods:**

Exosomes were isolated by differential ultracentrifugation from conditioned medium of HCC cells and identified by electron microscopy and Western blotting analysis. Hepatic stellate cells (HSCs) were treated with different concentrations of exosomes, and the activation of HSCs was analyzed by Western blotting analysis, wound healing, migration assay, Edu assay, CCK-8 assay and flow cytometry. Moreover, the different miRNA levels of exosomes were tested by real-time quantitative PCR (RT-PCR). The angiogenic ability of activated HSCs was analyzed by qRT-PCR, CCK-8 assay and tube formation assay. In addition, the abnormal lipid metabolism of activated HSCs was analyzed by Western blotting analysis and Oil Red staining. Finally, the relationship between serum exosomal miRNA-21 and prognosis of HCC patients was evaluated.

**Results:**

We showed that HCC cells exhibited a great capacity to convert normal HSCs to cancer-associated fibroblasts (CAFs). Moreover, our data revealed that HCC cells secreted exosomal miRNA-21 that directly targeted PTEN, leading to activation of PDK1/AKT signaling in HSCs. Activated CAFs further promoted cancer progression by secreting angiogenic cytokines, including VEGF, MMP2, MMP9, bFGF and TGF-β. Clinical data indicated that high level of serum exosomal miRNA-21 was correlated with greater activation of CAFs and higher vessel density in HCC patients.

**Conclusions:**

Intercellular crosstalk between tumor cells and HSCs was mediated by tumor-derived exosomes that controlled progression of HCC. Our findings provided potential targets for prevention and treatment of live cancer.

**Electronic supplementary material:**

The online version of this article (10.1186/s13046-018-0965-2) contains supplementary material, which is available to authorized users.

## Introduction

Hepatocellular carcinoma (HCC) is responsible for the third most common cancer-related death worldwide [1]. Importantly, the vast majority of HCC is caused by liver fibrosis or cirrhosis [2]. Liver fibrosis occurs when inactive liver fibroblasts or hepatic stellate cells (HSCs) become activated after liver injury and turn into collagen-producing cells [3]. In HCC, cancer-associated fibroblasts (CAFs) are loosely defined as HSCs found within the tumor mass [4]. These CAFs have been implicated as etiologic players both in the cancer genesis and homeostasis. These data suggest that liver fibrosis, or activated HSCs, plays a crucial role in the development of HCC, which may be similar to the role of CAFs in desmoplastic tumors.

How fibroblasts are activated in HCC remains controversial. Recent studies have revealed multiple potential origins, including activation of stellate cells or portal fibroblasts or transdifferentiation of hepatocytes through epithelial-mesenchymal transition (EMT) [5]. Stemmed from different origins, CAFs are highly heterogeneous, and they can be identified by different specific markers [6]. Among them, α-smooth muscle actin (α-SMA) is the most commonly used marker for CAFs [7]. Moreover, CAFs are believed to regulate the inflammatory microenvironment by expressing pro-inflammatory genes, such as IL-1β, IL-6, IL-8, TGF-β and CXCL12 as well as collagen [8, 9]. The crosstalk between tumor cells and CAFs has been extensively studied [10–12]. However, the mechanisms underlying the activation of HSCs by tumor cells remain largely unexplored in liver cancer.

An important type of cell-cell communication occurs through exosomes. These small, nanometer-sized (50–100 nM) vesicles of endocytic origin are released into the extracellular milieu by cells under physiological and pathological conditions, including antigen presentation and infectious agent transmission. Tumor exosomes are important mediators of the cross-talk between tumor cells and their microenvironment by sharing genetic information or functional proteins to modulate cellular behavior [13, 14]. Tumor cell-derived exosomes are involved in the regulation of EMT, tumor angiogenesis, tumor metastasis and radioresistance [15, 16].

It has been revealed that microRNA (miRNA) dysregulation greatly contributes to the activation of HSCs [17]. Interestingly, there is a significant overlap in the list of up-regulated or down-regulated miRNAs between HCC tumor tissues and normal tissues [18]. It has been demonstrated that secreted miRNAs can function in a paracrine manner in the surrounding microenvironment, promoting tumor development [19]. Meanwhile, studies have indicated that exosomes contain a high level of miRNAs, and exosomal miRNAs have been shown to contribute to immunomodulation, chemoresistance and metastasis in multiple types of tumor [20, 21]. However, it remains unclear how HSCs are activated through miRNA pathways.

In the present study, we aimed to classify the mechanisms underlying the activation of HSCs in liver cancer. Moreover, CAFs promoted a set of properties of vascular endothelial cells via increasing the secretion of angiogenic factors. The bilateral interaction between primary tumor cells and stromal cells further illuminated a new mechanism of tumor progression and offered new opportunities for potential therapeutic strategies targeting HCC development.

## Methods

### Specimens and primary cells

Human serum specimens and liver tissues were collected from healthy donors and HCC patients before or after resection in the Nanjing Drum Tower Hospital in Nanjing, China. All procedures were approved by the Ethical Committee of the Nanjing Drum Tower Hospital. Written informed content was obtained from every participant prior to study.

### Cell culture

The human liver cancer cell lines (97H, LM3 and Huh7), the vascular endothelial cell line HUVEC, and the HSC cell line LX2 were purchased from Cell Bank of Type Culture Collection of the Chinese Academy of Sciences (Shanghai Institute of Cell Biology) and maintained in DMEM (Gibco) supplemented with 10% FBS (Gibco). The normal liver cell line LO2 was purchased from Cell Bank of Type Culture Collection of the Chinese Academy of Sciences and maintained in RPMI-1640 medium (Gibco) supplemented with 10% FBS (Gibco). All cell lines were cultured at 37 °C in a humidified incubator containing 5% CO_2_. Cell lines were authenticated by short tandem repeats (STR) profiling and confirmed to be mycoplasma negative.

### Reagents and antibodies

Antibodies against CD63 (ab125011, 1:1000), α-SMA (ab32575, 1:100), FAP (ab207178, 1:1000), FSP (ab124805, 1:1000), GAPDH (ab8245, 1:10,000), CD9 (ab92726, 1:1000), CD81 (ab79559, 1:1000), FASN (ab22759, 1:500), ATP citrate lyase (EP704Y, 1:1000) and phospho-ATP citrate lyase (T447/S451) (ab53007, 1:1000) and USP2 (ab66556, 1:500) were purchased from Abcam (Cambridge, MA, USA). Antibodies against phosphor-PTEN (Ser380/Thr382/383) (9554S, 1:1000), total PTEN (7960 T, 1:1000), PDK1 (3062 T, 1:1000), phospho-PDK1 (Ser241) (3438 T, 1:1000), phospho-Akt (Ser473) (4060 T, 1:1000) and AKT (4691 T, 1:1000) were supplied by Cell Signaling Technology (Beverly, MA, USA). AKT inhibitor MK-2206 was provided by Selleck (Houston,USA).

### Western blotting analysis

Whole-cell protein extracts were homogenized in lysis buffer and centrifuged at 12,000 r.p.m. for 15 min. Protein concentrations were determined by bicinchoninic acid (BCA) assay. After immunoblotting, the proteins were transferred onto nitrocellulose membranes, followed by incubation with specific antibodies. The immunocomplexes were then incubated with the fluorescein-conjugated secondary antibody, and immunoreactive bands were visualized by an Odyssey fluorescence scanner (Li-Cor, Lincoln, NE).

### RNA interference

siRNAs and mimics of indicated miRNAs were obtained by RiboBio Company (Guanngzhou, China). The sequences of siRNAs and miRNA mimics referred above were listed in Additional file 1: Table S1. Transfection with siRNAs and miRNAs was completed using riboFECT™ CP (RiboBio) according to the manufacturer’s instructions.

### Animal studies

To examine the roles of exosomes in CAFs, 1 × 10^6^ Huh7 cells were intravenously injected into male nude mice through the tail vein (Chinese Science Academy, Shanghai, China). Subsequently, mice were randomly divided into groups and intravenously injected with equal numbers of exosomes from different tumor cells twice a week for 1 month. For xenograft assays, 1 × 10^6^ Huh7 cells and 1 × 10^6^ LX2 cells were injected subcutaneously into the right side of each male nude mouse (Chinese Science Academy). The sizes of tumors (length × width: 2 × 0.5) were measured at the indicated time points, and tumors were obtained at 4 weeks after injection. All animal experiments were approved by the University Committee on Use and Care of Animals of Nanjing Drum Tower Hospital.

### Cell viability assay, migration assay and wound-healing assay

For cell viability assay, Cell Counting Kit 8 (CCK-8) assay (Dojindo Laboratories, Kumamoto, Japan) was used to assess cell viability according to manufacturer’s instructions. For migration assay, 5 × 10^4^ LX2 cells were plated into 24-well transwell plates with inserts (Corning). The medium in inserts was free of FBS, whereas the medium outside the inserts was supplemented with 10% FBS. To detect exosome function, equal quantities of tumor-derived exosomes were added into the inserts. After 24 h, the cell inserts were fixed and stained according to manufacturer’s protocols. Representative fields were photographed, and the number of migrated cells per field was counted. For wound-healing assay, equal numbers of LX2 cells were plated into six-well plates. Then the cell monolayers were wounded with a pipette tip to draw a gap on the plates. After treated with tumor-derived exosomes, fibroblasts that migrated into the cleared section were observed under microscope at the specific time points.

### RNA extraction and qRT-PCR

HSC RNA was extracted from snap-frozen liver tissues with TRIzol™ reagent (Life Technologies, USA) according to the manufacturer’s instructions. Briefly, cells in six-well plate were homogenized in 1 mL TRIzol™ reagent at room temperature. Then 200 μL trichloromethane was added and mixed thoroughly, followed by centrifugation at 12,000×g for 15 min at 4 °C. The upper clear phase was collected and added with 500 μL isopropanol, followed by centrifugation at 12,000×g for 10 min at 4 °C. The liquid supernatant was discarded, and the pellet was washed with 75% ethanol. The RNA was re-suspended in 50 μL RNase-free DEPC-water and stored at − 80 °C.

Reverse transcription was performed with PrimeScript™ RT Master Mix (Takara, Japan) according to the manufacturer’s instructions. qRT-PCR was performed using TB Green™ Premix Ex Taq™ (Takara, Japan) on an ABI PRISM 7500 real-time PCR System (Applied Biosystems, USA). Primers used for qPCR are shown in Additional file 1: Table S1. The relative expression levels of mRNAs were calculated with 2^–ΔΔCt^ method. GAPDH was selected as the housekeeping gene.

miRNeasy Mini Kit (Qiagen) was used to extract RNA from exosomes in plasma/medium according to the manufacturer’s instructions, and cel-miR-39 (Takara) was added into each sample at a final concentration of 10 pmol/μL acting as external reference. Total RNA was stored at − 80 °C for subsequent experiments.

Reverse transcription and qRT-PCR for exosomal miRNA, as well as internal reference U6 were performed using miRNA RT-PCR Quantitation Kit (Qiagen) according the manufacturer’s instructions. Briefly, after an initial denaturation step at 95 °C for 3 min, the amplifications were carried out with 40 cycles at a melting temperature of 95 °C for 15 s, and an annealing temperature of 62 °C for 34 s. The relative expression levels of exosomal miRNAs were calculated with 2^–ΔΔCt^ method.

### Oil red staining

Cells were fixed in 10% formalin, washed with 60% propylene glycerol, and then stained with 0.5% oil red O (Sangon Bio) in propylene glycerol for 10 min at 60 °C. The red lipid droplets were visualized by microscopy.

### Immunohistochemistry and in situ hybridization analysis

For immunohistochemistry, the slides were incubated with above-mentioned primary antibodies, followed by incubation with horseradish peroxidase-conjugated secondary antibodies (Santa Cruz Biotechnology). Finally, the staining processes were performed with diaminobenzidine colorimetric reagent solution (Dako, Carpinteria, USA) and hematoxylin (Sigma Chemical Co., USA). For in situ hybridization analysis, hsa-miRNA-21 miRCURY LNA detection probe (Exiqon, Denmark) was used, and the total staining processes were carried out according to manufacturer’s protocols. Images were captured with Aperio ScanScope AT Turbo (Aperio, USA) and assessed with image-scop software (Media Cybernetics, Inc.).

### Immunofluorescence analysis

Immunofluorescence analysis was performed according to previously established protocols. Cells were seeded into 24-well dishes and fixed by 4% paraformaldehyde 24 h later. Fixed cells were stained with α-SMA and FAP antibodies (mentioned above), followed by incubation with FITC-conjugated anti-mouse IgG and Cy3-conjugated anti-rabbit IgG (Abcam). Representative images were detected by fluorescence microscopy (Leica, German), and data were processed via Photoshop.

### Tube formation assay

EC matrix was thawed at 4 °C overnight, and required wells of pre-chilled 96-well plates were coated with 50 μL diluted EC matrix and incubated at 37 °C for 1 h to solidify. Subsequently, 150 μL of HUVEC cells (1 × 10^4^) with different conditional media were added to the solidified matrix and incubated at 37 °C for 12 h. Endothelial cell formation was observed using a microscope. Focus was placed on distinct areas, and the tubes formed were counted according to the kit procedure.

### Isolation and analysis of exosomes

For exosome isolation, equal numbers of different cells were transplanted into 10-cm plates and maintained in fresh DMEM supplemented with serum, which was depleted of exosomes by centrifugation at 12,000×g overnight. After 48 h, CM was collected and filtrated through 0.22-μm filters (Millipore, USA). Exosomes in CM or serum samples were isolated by ultracentrifugation according to the standard methods described previously [22]. Ultracentrifugation experiments were performed with Optima MAX-XP (Beckman Coulter, USA). Exosomes were observed by Philips CM120 BioTwin transmission electron microscope (FEI Company, USA).

### Exosomes tracing

For exosome-tracing experiments, tumor cells were pre-treated by PKD67 (Beyotime, China), and exosomes in CM were obtained as described above. After incubation with recipient cells that were pre-treated with FAP antibody and DAPI (Beyotime), exosomes were observed by fluorescence microscope (Leica, German).

### Transmission electron microscopy

The obtained exosomes were fixed with fixative buffer containing 2% paraformaldehyde and 2.5% glutaraldehyde in 0.1 M PBS. After embedding, samples were cut into 0.12-μm sections and stained with 0.2% lead citrate and 1% uranyl acetate. The images were detected by a JEOL TEM-2000 EX II (JEOL, Tokyo, Japan).

### Cell cycle analysis

HSCs were seeded into six-well plates at a density of 1.5 × 10^5^ cells/well overnight, and the cells were conditionally cultured for 24 h. Cell cycle was detected using cell cycle analysis kit (Thermo Fisher Scientific, USA) according to the manufacture’s protocols and measured by flow cytometry.

### Collagen contraction assay

A total of 2 × 10^5^ HSCs were suspended in 100 μL DMEM. Then the cell suspension was mixed with 100 μL of collagen mix containing 68.75 μL DMEM, 0.72 μL 1 N NaOH and 31.25 μL type 1 rat tail collagen (Corning), added to one well of 6-well plates and allowed to solidify for 45 min at 37 °C. After incubation with medium containing HCC-derived exosomes, the gels were photographed by digital camera.

### Statistical analysis

Data analysis was performed using the SPSS software version 16. Each experiment was carried out in triplicate at least, and all results were presented as mean ± SD. χ2-Test and Student’s t-test were used to assess statistical significance. Kaplan–Meier analysis and log-rank tests were applied for survival analysis. A *p* value of < 0.05 was considered as statistically significant.

## Results

### Tumor-derived exosomes regulate activation of HSCs

**Fig. 1:**
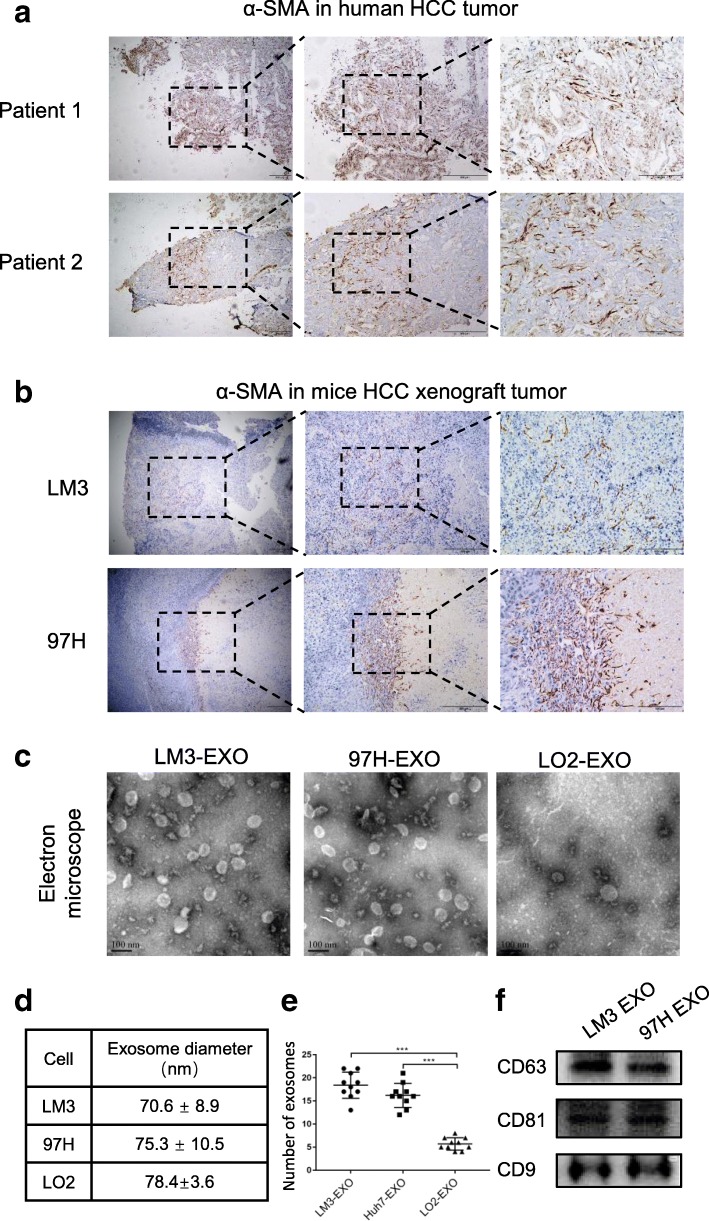
Tumor-derived exosomes activated HSCs. **a**, **b** Representative images of α-SMA staining in primary HCC and in orthotopically implanted tumors in mice. **c**, **d**, **e** Exosomes released by different cells were detected by electron microscopy. **f** Immunoblotting assay of indicated proteins in exosomes from different cancer cells. Each experiment was performed three times independently, and results are presented as mean ± s.d. Student’s t-test was used to analyze the data. (*p < 0.05; **p < 0.01; ***p < 0.001)

CAFs have been demonstrated to actively participate in the tumor invasion. Based on the high expression of α-SMA, the most effective marker, similar results were observed in tissues from HCC patients (Fig. 1a) and in orthotopically implanted tumors in mice (Fig. 1b). Compared with the high expression of α-SMA in HCC tissue, the expression of α-SMA was negative in normal tissue (Additional file 2: Figure S1a). Therefore, it was very important to further understand the activation of CAFs caused by tumor cells. Tumors mainly induce systemic changes through exosome secretion [23]. However, whether exosomes participate in the activation of HSCs remains largely unexplored. In the present study, two liver cancer cell lines (97H and LM3) and one liver cell line (LO2) were employed to clarify this question. First, we purified exosomes from tumor cell-conditioned medium through ultracentrifugation, a standard exosome isolation method. The cup-shaped structure, size and number of isolated exosomes were identified by electron microscopy (Fig. 1d-f). Intriguingly, we demonstrated that much more exosomes were secreted from HCC cells compared with LO2 cells (Fig. 1e). In addition, the detection of characteristic CD63, CD81 and CD9 further verified that the isolated particles were exosomes (Fig. 1f).

**Fig. 2:**
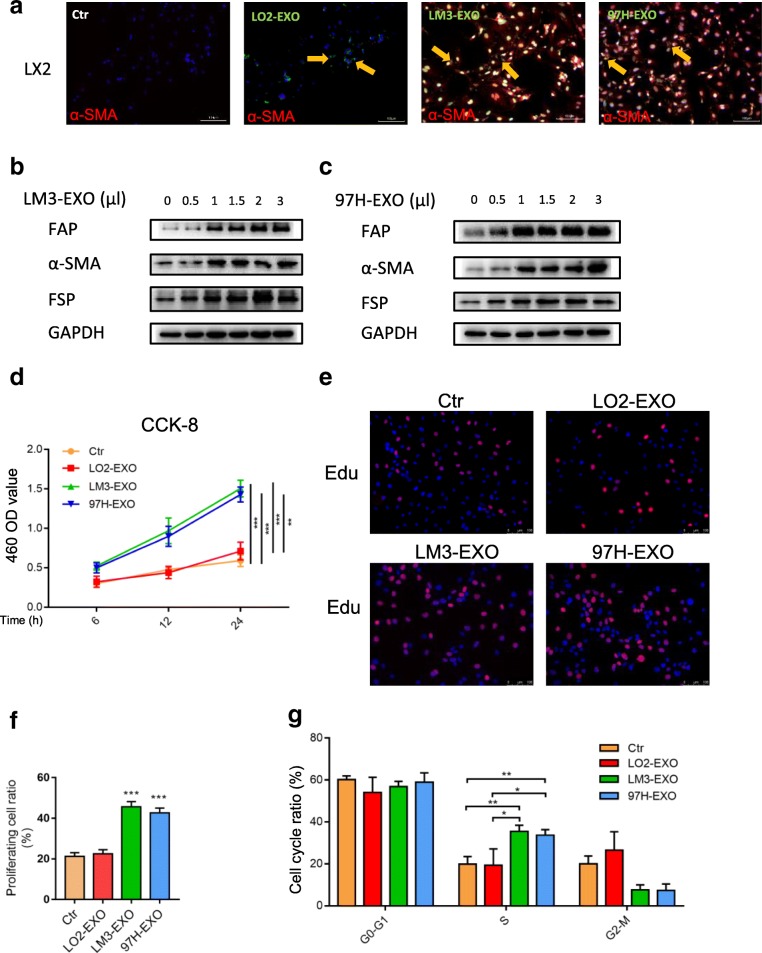
Tumor-derived exosomes activated HSCs in vitro. **a** Immunofluorescence imaging showed the delivery of 97H-labeled exosomes (green) to α-SMA-labeled HSCs (red). Yellow arrows represented delivered exosomes. **b** and **c** HSCs were activated by HCC cell-derived exosomes in a concentration-dependent manner. CCK8 assay (**d**), Edu (**e** and **f**) and flow cytometry assays (**g**) of HSCs treated with equal quantities of exosomes derived from different liver cancer cells or blank control. Experiments were performed at least in triplicate, and results are shown as mean ± s.d. Student’s t-test was used to analyze the data (*p < 0.05; **p < 0.01; ***p < 0.001)

**Fig. 3:**
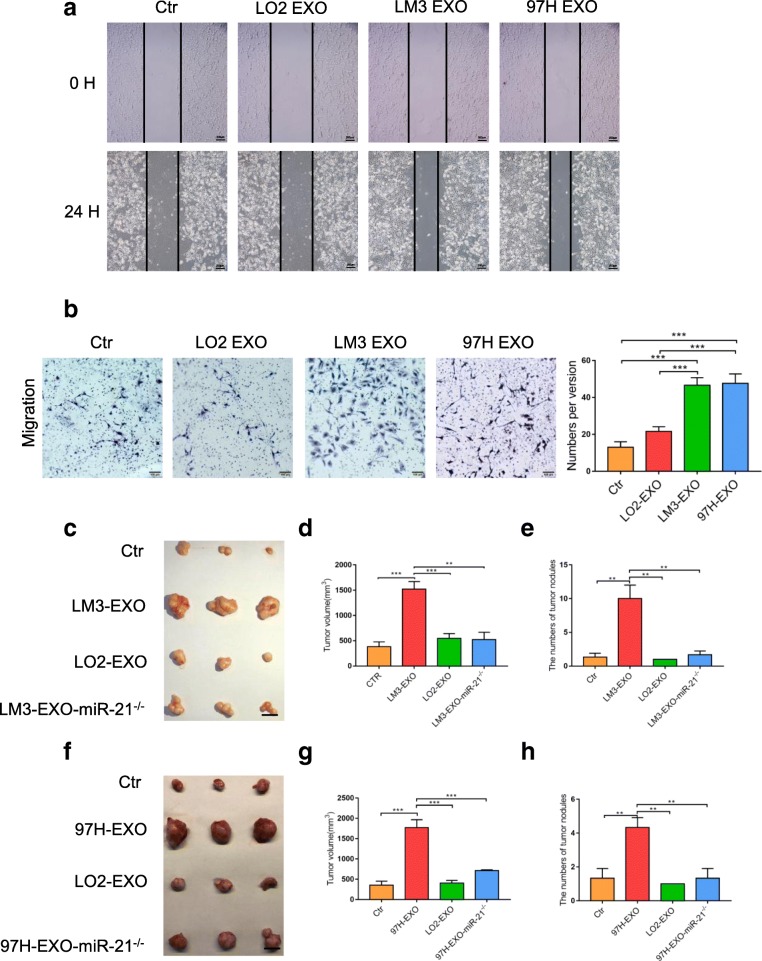
Tumor-derived exosomes activated HSCs in vitro. Wound-healing assays (**a**) and migration assay (**b**) of HSCs treated with equal quantities of exosomes derived from different liver cancer cells or blank control. **c** – **h** Xenograft assays of Huh7 with indicated treatments were performed on nude mice. Representative tumors, tumor volume and number of tumor nudes were shown. Experiments were performed at least in triplicate, and results are shown as mean ± s.d. Student’s t-test was used to analyze the data (NS, not significant; *p < 0.05; **p < 0.01; ***p < 0.001)

As a human cell line of HSCs, LX2 was selected as normal HSCs in our study. To identify the delivery of exosomes, we labeled the tumor-derived exosomes with PKH67 (green). After incubation, immunofluorescence imaging showed the presence of green spots in recipient HSCs, suggesting that labeled exosomes released by different cells were delivered to HSCs (Fig. 2a). Meanwhile, the expression of α-SMA was high, suggesting that HSCs were activated by tumor cell-derived exosomes, not by liver cell-derived exosomes (Fig. 2a). Western blotting analysis revealed that HSCs were activated by HCC cell-derived exosomes in a concentration-dependent manner (Fig. 2b and c). Moreover, we showed that HCC cell-derived exosomes were positively correlated with the activation of HSCs. Since the increased cell proliferation is a manifestation of activated HSCs, we examined whether HCC cell-derived exosomes affected the proliferation of HSCs. CCK-8 assay indicated that HCC cell-derived exosomes significantly promoted the cell growth in a time- dependent manner (Fig. 2d). To further evaluate the different abilities of educating HSCs among the exosomes derived from different cells, Edu incorporation assays and flow cytometry assays showed that the proportion of HSCs in the S phase was increased, suggesting that the cell proliferation was increased under the stimulation of HCC cell-derived exosomes (Fig. 2e-g and Additional file 3: Figure S2a). Wound-healing assay further confirmed that HCC cell-derived exosomes could remarkably improve the migration ability of HSCs (Fig. 3a). Migration assay showed that more HSCs migrated in the 97H-EXO group and LM3-EXO group compared with the control group and LO2 group, respectively (Fig. 3b). The contraction abilities of HSCs were markedly enhanced after treatment with HCC cell-derived exosomes compared with LO2 cell-derived exosomes or control (Additional file 3: Figure S2b). Moreover, high expression levels of proimflammatary factors were found in the activated HSCs (Additional file 3: Figure S2c). In addition, intravenous exosomes from LM3 or 97H markedly contributed to the growth of subcutaneous implanted tumor (mixed injection of HCC-Huh7 and LX2) in nude mice, with more nodules detected at the same time (Fig. 3c-h). Immunofluorescence assay indicated that PKH67-labeled exosomes (green) existed in CAFs which expressed FAP (red) (Additional file 4: Figure S3a). Ki67 staining showed that the proliferation of CAFs was increased under the stimulation of HCC exosomes (Additional file 5: Figure S4a). Taken together, the above-mentioned results suggested that exosomes derived from highly metastatic HCC cells greatly contributed to the activation of HSCs to foster CAFs.

### MiRNA-21 in exosomes mediates activation of HSCs

**Fig. 4:**
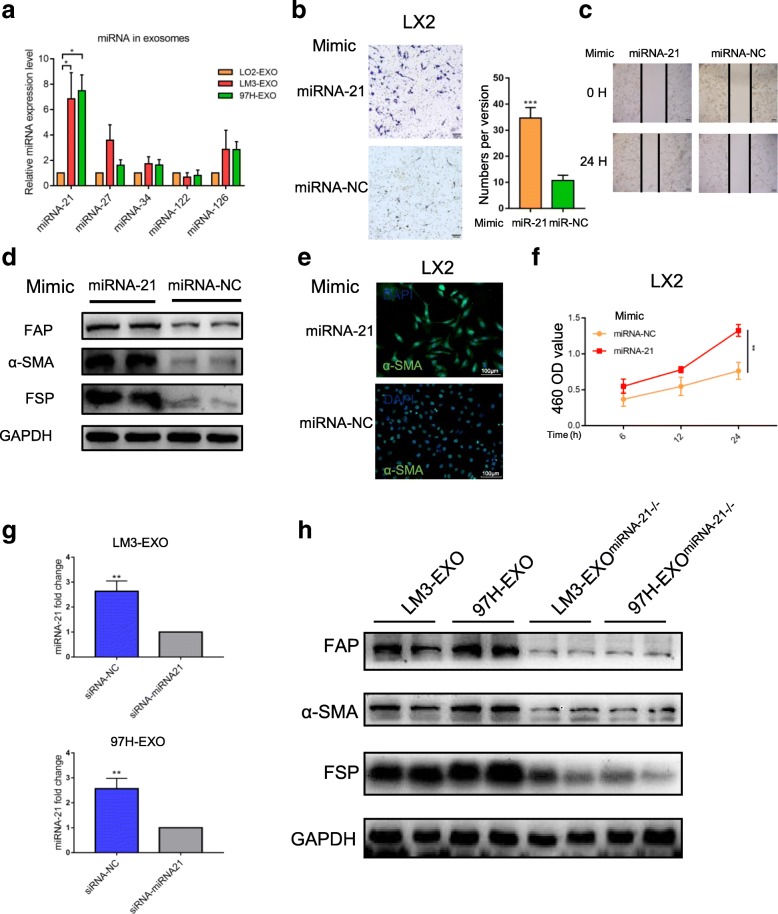
Exosomal miRNA-21 is characteristically secreted by liver cancer cells and mediates HSCs activation. **a** Expression of exosomal miRNAs from different cancer cells was presented. Wound-healing assay (**b**), migration assay (**c**), Western blotting assay (**d**), immunofluorescence assay (**e**) and CCK-8 assay (**f**) of HSCs transfected miRNA-21-mimic or normal control. Representative images were shown. **g** qPCR of miRNA-21 in HCC-derived treated with miRNA-21 inhibitor exosomes. **h** Western blotting assay of HSCs transfected miRNA-21 inhibitor. Experiments were performed at least in triplicate, and results are shown as mean ± s.d. Student’s t-test was used to analyze the data (NS, not significant; *p < 0.05; **p < 0.01; ***p < 0.001)

We next explored how tumor-derived exosomes activated HSCs. MiRNAs encapsulated in exosomes are abundant, playing an important role in cell-cell communication [24]. Therefore, we hypothesized that tumor-derived exosomal miRNAs mediated the activation of HSCs. Five types of miRNAs, which are closely related to liver cancer [25], were selected to verify this hypothesis. Quantitative reverse-transcriptase PCR (qRT-PCR) analysis further confirmed the elevated expression of miRNA-21 in both cancer cell-derived exosomes (Fig. 4a) and HCC cells (Additional file 6: Figure S5a). When the HCC cell-derived exosomes were added into the culture medium of LX2, the expression of miRNA-21 in HSCs was increased, coupled with the activation of HSCs (Additional file 7: Figure S6a). Furthermore, miRNA-21 mimic also contributed to potential activation of HSCs (Fig. 4b-f, Additional file 7: Figure S6b-e). To further investigate the role of miRNA-21, HCC cells were transfected with miRNA-21 inhibitor. After the transfection, the expression of miRNA-21 in HCC cell-derived exosomes was reduced (Fig. 4g). As expected, the effect of miRNA-21 on HSC activation was abolished by siRNA-miRNA-21 (Fig. 4h, Additional file 8: Figure S7 and Additional file 9: Figure S8). Collectively, these findings revealed that tumor-derived exosomal miRNA-21 mediated the activation of HSCs.

### Exo-MiRNA-21 activates HSCs via PTEN/PDK1/AKT axis and lipogenesis

**Fig. 5:**
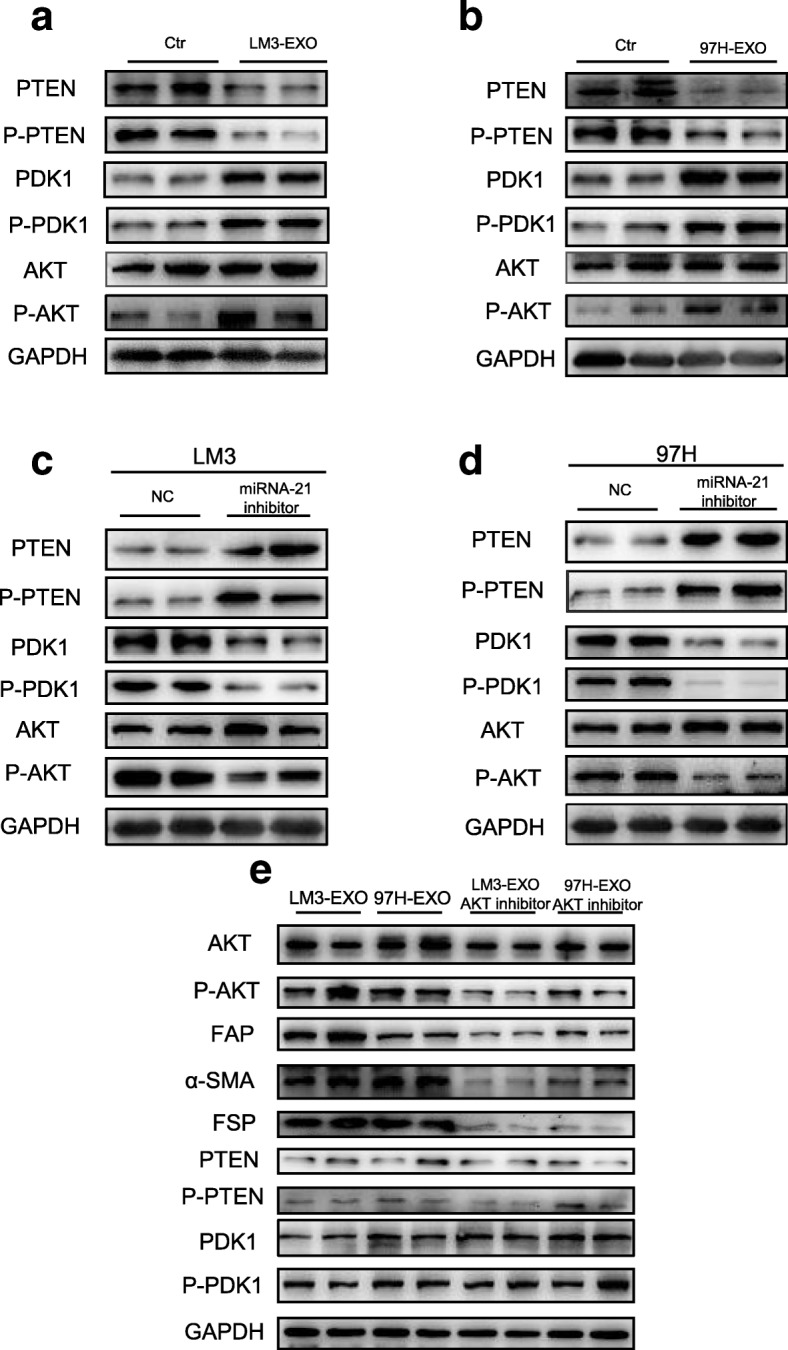
Exosomal miRNA-21 activates HSCs via PTEN/PDK1/AKT signaling axis. **a**, **b** Immunoblotting assays of indicated proteins in HSCs treated with control or exosomes from different tumor cells. **c**-**e** Western blotting assays of indicated proteins in HSCs with indicated treatments. Each experiment was performed in triplicate, and data are presented as mean ± s.d. Student’s t-test was used to analyze the data (*p < 0.05; **p < 0.01; ***p < 0.001)

Previous experiments have shown that PTEN (gene of phosphate and tension homology deleted on chromsome ten), a well known tumor-suppressor gene, is the target for miRNA-21 [26]. Therefore, we assessed the PTEN expression in our experimental models. Figure 5a and b exhibit that exosomes of HCC cells reduced phosphorylated level of PTEN, along with the increased expression of its downstream inhibitory protein PDK1 (3-phosphoinositide-dependent protein kinase-1) compared with the control group. Furthermore, miRNA-21 suppression also promoted the PTEN phosphorylation and reduced the PDK1 phosphorylation (Fig. 5c and d). Meanwhile, PTEN is a negative regulator of v-akt murine thymoma viral oncogene homolog (Akt) pathway [26]. Akt pathway is believed to be involved in the regulation of HCC [27]. To examine whether AKT pathway was regulated by miRNA-21 in activated HSCs, we examined the phosphorylation of Akt. Figure 5a and b show that the phosphorylation of AKT was increased in HCC-exosome-miRNA-21-activated HSCs. The above-mentioned results indicated that PTEN/PDK1/Akt signaling might play a role in miRNA-21-induced stimulation in HSCs. When the MK-2206, an efficient inhibitor of AKT, was added to the culture medium of HSCs, the activation of HSCs caused by cancer cell-derived exosomes was reversed (Fig. 5e and Additional file 8: Figure S7 and Additional file 9: Figure S8). These results confirmed that HCC-exosome-miRNA-21 regulated the activation of HSCs through PTEN/PDK1/Akt pathway.

**Fig. 6:**
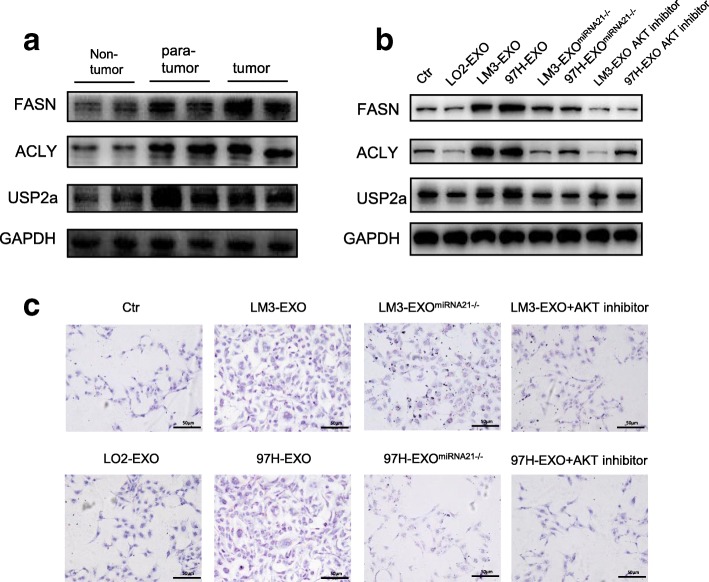
HCC derived exosomes induced abnormal lipid metabolism. **a**, **b** Western blotting assays of lipid metabolism related proteins in HCC patients or HSCs with different stimulations. **c** Oil Red staining assay showed the abnormal lipid accumulation in HSCs with indicated treatments. Each experiment was performed in triplicate, and data are presented as mean ± s.d. Student’s t-test was used to analyze the data (*p < 0.05; **p < 0.01; ***p < 0.001)

Aberrant lipogenesis has been linked to metabolic abnormalities, such as diabetes, obesity and the metabolic syndrome as well as cancer [28]. In the latter disease, unconstrained lipogenesis is necessary to maintain a constant supply of lipids and lipid precursors to fuel membrane production and lipid-based post-translational modification of proteins in a context of elevated proliferation. At the molecular level, exacerbated lipogenesis is reflected by the coordinately increased activity and expression of lipogenic enzymes in neoplastic cells, such as ATP citrate lyase (ACLY) and fatty acid synthase (FASN). Previous study has demonstrated that activated AKT also regulates the enzymatic activities of ACLY and FASN [29]. Therefore, we investigated the levels of lipogenic pathway enzymes, ACLY and FASN, by immunoblotting. We found that a progressive induction of FASN and ACLY occurred in tumor and para-tumor (Fig. 6a). Previously, it has been shown that ubiquitin-specific protease 2a (USP2a) sustains FASN activity by impeding its ubiquitin-dependent degradation [30]. Therefore, we determined the USP2a level in our samples. Our data showed that the USP2a level was up-regulated in tumor and para-tumor as well (Fig. 6a). Considering that the CAFs are mostly localized on the periphery of the tumor, we then assessed the activation of HCC cell-derived exosomes in HSCs by immunoblotting. Compared with the control group, cancer cell-derived exosomes were significantly and positively correlated with lipogenesis since the levels of ACLY, FASN and USP2a were increased in exosome-challenged HSCs (Fig. 6b). Moreover, the activation of HSCs was abolished by miRNA-21 inhibitor and AKT inhibitor (Fig. 6b). To further examine whether cancer cell-derived exosomes were associated with lipogenesis in HSCs, we assessed the severity of steatosis by oil eed assay. Compared with control group and LO2-EXO group, we observed that LM3-EXO and 97H-EXO led to increased levels of lipid staining in HSCs, whereas the staining was significantly lower in miRNA-21 inhibitor group and AKT inhibitor group (Fig. 6c). Collectively, the present data indicated that there was a positive correlation between cancer cell-derived exosomes and lipid contents in HSCs.

### Activated HSCs promote angiogenesis

**Fig. 7:**
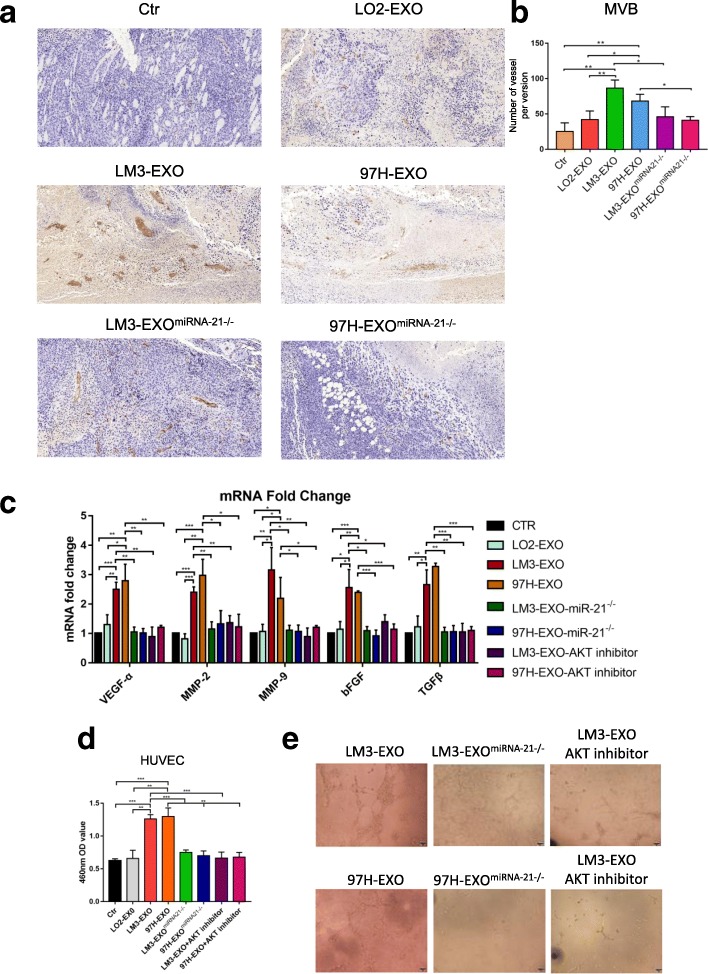
Activated HSCs promote angiogenesis **a**, **b** Immunohistochemistry staining of CD31 staining in subcutaneously implanted tumor under different conditions. **c** qRT-PCR assay indicated gene expression levels of HSCs treated with exosomes derived from different cells in the presence of miRNA-21 inhibitor or AKT inhibitor. Experiments were performed at least in triplicate. **d**, **e** The proliferation of HUVECs with exosomes derived from different cells in the presence of miRNA-21 inhibitor or AKT inhibitor was tested by CCK-8 assay and tube formation assay. Data are mean ± SEM from three independent experiments, *p < 0.05, **p < 0.01, ***p < 0.001 by unpaired Student’s t-test

The transition from pre-angiogenic to macroscopic metastasis is a rate-limiting step in the hepatic metastatic cascade [31], which can be used to identify the development of an angiogenic phenotype as a key factor for metastatic progression within the liver. CAFs can promote cancer progression through various mechanisms, including the stimulation of angiogenesis. Under certain circumstances, CAFs promote tumor angiogenesis. However, they have not been linked to angiogenesis, especially in the hepatic metastatic microenvironment. We assessed the vasculature in HCC mice using immunohistochemistry. In subcutaneously implanted HCC tumor composed of Huh-7 cells and LX2 cells under the stimulation of cancer cell-derived exosomes, vascular density was significantly higher than that of the control group, where as the inhibitor of the miRNA-21 reversed the tumor volume (Fig. 7a and b). At the same time, we found that CAFs were significantly closer to vessels than other cells in the tumor by immunofluorescence (Additional file 10: Figure S9), indicating that they resided in proximity to the developing tumor vasculature. This would be the ideal position, in which CAFs could promote angiogenesis through deposition of angiogenic factors in the vascular basement membrane. In order to determine whether CAFs expressed factors capable of promoting angiogenesis, we compared the mRNA expression profiles of CAFs activated by cancer cell-derived exosomes and normal HSCs. The expressions of VEGF-α, MMP2, MMP9, bFGF and TGF-β were all increased from CAFs of human HCC cancer cell lines compared with the normal HSCs (Fig. 7c). In vitro experiment showed that when vascular endothelial cells were co-cultured with HSCs stimulated by HCC cell-derived exosomes, the proliferation of cells was improved (Fig. 7d), and the ability of cells to form tubular structures was significantly enhanced (Fig. 7e). When the miRNA-21 inhibitor and AKT inhibitor were added, the angiogenesis ability of CAFs was remarkably reduced (Fig. 7c and d). The above-mentioned results suggested that HCC cell-derived exosomes could activate CAFs, leading to improved tumor growth through angiogenesis.

### MiRNA-21 correlates with prognosis of HCC patients

**Fig. 8:**
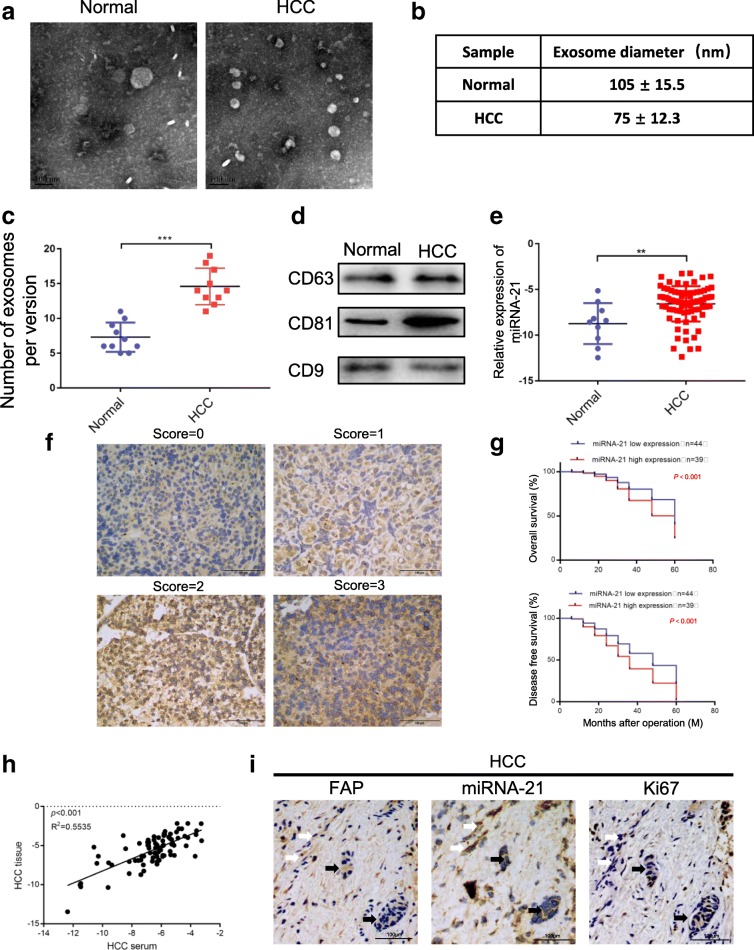
miRNA-21 is associated with HCC progression. **a** – **d** Exosomes in normal and HCC serums were detected by electron microscopy. **e** miRNA-21 expression level in serum exosomes from healthy donors and primary HCC patients. Data are presented as mean ± s.d. Student’s t-test was used to analyze the data. **p < 0.01. **f** In situ hybridization assay of HCC samples and scores of miRNA-21 level. Representative images were shown. **g** Kaplan–Meier plots of overall survival and disease-free survival of 83 HCC patients, stratified by expression of miRNA-21. Survival data were analyzed by the Kaplan−Meier method and log-rank test. **h** The correlation analysis between expression of miRNA-21 in serum and expression of miRNA-21 in HCC was detected. **i** In situ hybridization of miRNA-21 in combination with IHC staining of HSCs markers (FAP) and proliferation markers (Ki67) on serial sections of human HCC tissues. White arrows indicate CAFs; black arrows indicate vessels

To extend current knowledge to HCC patients, we first examined serum exosomes isolated from HCC patients and healthy controls by electron microscopy and Western blotting analysis (Fig. 8a-d). Consistent with previous findings, a substantially increased excretion of serum exosomes was detected in HCC patients compared with the healthy controls [32]. Moreover, we investigated the expression of exosomal miRNA-21 in different serum samples (10 healthy controls and 85 HCC patients). Figure 8e shows that the serum expression of exosomal miRNA-21 was increased in HCC patients compared with the healthy controls. To further determine the correlation between miRNA-21 and clinical pathological features, the HCC patients were divided into two groups (low and high) according to the expression scores of miRNA-21 (Fig. 8f). As shown in Fig. 8g, high miRNA-21 expression (score = 2 and 3) could well predict the poor overall survival and poor disease-free survival. In HCC patients, correlation analysis showed that there was a positive correlation between miRNA-21 in serum and miRNA-21 in tissue (Fig. 8h). In addition, high miRNA-21 expression was closely related to high expressions of fibroblast marker (α-SMA), cell proliferation (Ki67 staining) and vessels (Fig. 8i). Taken together, these data showed that miRNA-21 was differentially expressed in liver cancer, and its high expression level in HCC tissues predicted poor outcome.

**Fig. 9:**
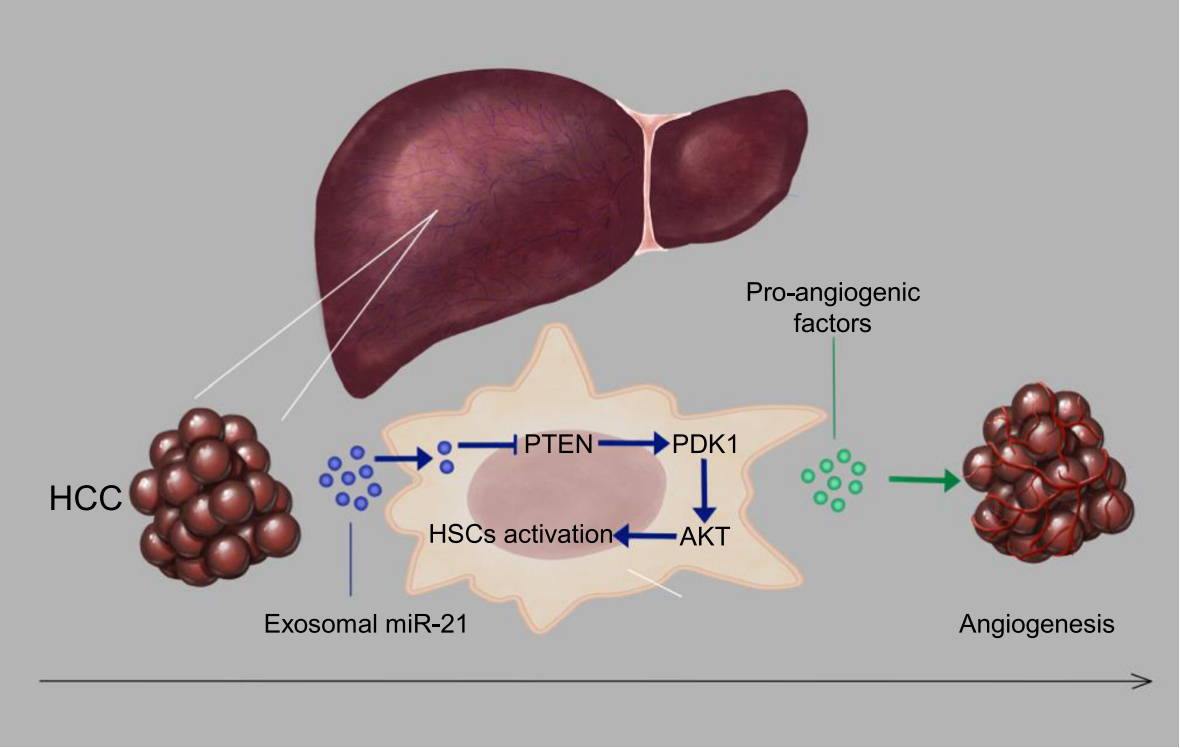
Proposed schematic diagram of HCC exosomal miR-21-mediating HSCs activation to promote angiogenesis of HCC

In summary, our results showed that tumor-derived exosomal miRNA-21 could convert HSCs to CAFs via down-regulating PTEN and activate PDK1/AKT signaling pathway to promote angiogenesis. Our findings also indicated that tumor-derived exosomal miRNA-21 played an important role in intercellular communication for fostering an inflammatory microenvironment and promoting tumor invasion through angiogenesis (Fig. 9).

## Discussion

As a dynamic system orchestrated by intercellular communications, tumor microenvironment is responsible for tumor progression and metastasis. Therefore, it is necessary to study the interaction between tumor and stroma mediated by exosomes. In our study, we first analyzed the different profiles of exosomal miRNAs between HCC cells and normal liver cells. Then, we identified that miRNA-21 was directly transferred from tumor cells to HSCs in tumor parenchyma via exosomes, and miRNA-21 could convert HSCs to CAFs by down-regulating its target PTEN to activate PDK1/AKT signaling pathway. In addition, CAFs promoted tumor development by angiogenesis through secreting IL-6 and IL-8. The crosstalk between tumor cells and HSCs further elucidated the molecular mechanism of HCC invasion, and explained why liver cancer was highly invasive. Furthermore, our data indicated that high miRNA-21 expression in serum exosomes was correlated with low survival rate, holding important implications for efficient prevention and therapeutic strategies. The involvement of miRNAs in cancer shows that the expressions of several miRNAs are dysregulated in neoplastic tissues [33]. The identification of several targets of miRNAs, which are actually classical oncogenes or tumor suppressors, has led to the widely accepted idea that miRNAs play pivotal roles in cancer initiation, progression and metastasization [34]. In another elegant study employing K-ras G12D non-small-cell lung cancer (NSCLC) mouse model, the results show that incidence of lung tumors is significantly high in miRNA-21-overexpressing mice. Consistent with this finding, deletion of miRNA-21 has resulted in suppression of Kras-driven transformation in vitro and tumor development in vivo [35]. Our data demonstrated that tumor-derived exosomal miRNA-21 converted HSCs to CAFs in HCC. Moreover, stimulation of exosomal miRNA-21 from HCC cells had a positive correlation with tumor volume in nude mice. Meanwhile, high miRNA-21 expression in HCC predicted a poor outcome. Previous studies have mainly focused on miR-21 overexpression in cancer cells, which promotes cellular proliferation, evasion of apoptosis, EMT and invasion. However, more and more attention has been paid to the role of miR-21 in CAFs. In a study on colorectal cancer, miRNA-21 expression is increased in stromal cells compared with normal tissues, and the ectopic expression of miR-21 drives the trans-differentiation of fibroblasts into myofibroblasts and increases invasion in vitro [36]. MiRNA-21 expression in lung fibroblasts may trigger fibroblast trans-differentiation into CAFs, supporting cancer progression. Furthermore, patients with miR-21-high CAFs exhibit lower survival compared with those with miR-21-low CAFs [37]. In summary, CAFs may further secrete exomal-miR21 to promote HCC progression. Future work will be required to study the role of miRNA-21 in liver cancer.

PTEN stands for phosphatase and TENs in homolog deleted on chromosome 10, and it is a classical tumor suppressor gene located in the 10q23 region of chromosome 10 encoding for a 403-amino acid multifunctional protein (predicted MW 47 kDa), which possesses lipid and protein phosphatase activities [38, 39]. PTEN functions as a classical tumor suppressor, and it is mainly involved in the homeostatic maintenance of the AKT cascade [40]. PI3K, a lipid kinase activated by receptor tyrosine kinases, G protein-coupled receptors and RAS activation, converts the lipid second messenger phosphatidylinositol 4,5-bisphosphate (PIP2) to phosphatidylinositol 3,4,5-trisphosphate (PIP3). PIP3 recruits PDK1 and AKT to the plasma membrane, where AKT is phosphorylated on Thr308 by PDK1. By dephosphorylating PIP3 to PIP2, PTEN reverses the action of PI3K, thereby hampering all downstream functions controlled by the AKT pathway, such as cycle progression, lipid synthesis and stimulation of angiogenesis. We found that decreased PTEN expression disturbed by HCC cell-derived exosomes increased phosphorylation levels of PDK1 and AKT, regulated lipid metabolism and promoted the release of angiogenic substances (VEGF, MMP2, MMP9, bFGF and TGF-β). PTEN also controls cell-cycle progression by decreasing the level of cyclin D1 in the nucleus and regulates cellular senescence. Consistent with previous studies, our results indicated that HSCs with low expression of PTEN under the stimulation of HCC cell-derived exosomes also showed high level of cyclin D1 and increased proportion of cells in the S phase.

Of the factors that were found to be induced by CAFs after co-culture with HCC cell-derived exosomes, the up-regulation of VEGF, MMP2, MMP9, bFGF and TGF-β was especially relevant, since these factors are known to promote cancer growth, invasion and angiogenesis through autocrine or paracrine signaling [41–43]. Additionally, these cytokines are associated with advanced stages of breast cancer and a poor clinical outcome. VEGF can also stimulate angiogenesis and it is significantly associated with a poor survival [44]. MMP2 and MMP9 are known multifunctional proteins. Data from diverse experimental models have indicated that these proteases affect cellular activities, including proliferation and survival, gene expression as well as multiple aspects of inflammation. Previous study has reported associations between high expression of MMP2 or MMP9 and tumor aggressiveness in liver cancer [45]. As a highly angiogenic molecule, bFGF is of particular interest since it is capable of promoting both the proliferation and migration of endothelial cells in various tumor models, and it can function synergistically with other factors to promote angiogenesis [46]. In summary, we found that HCC cell-activated CAFs could induce the expressions of several factors related to angiogenesis and tumor progression in HCC.

Aberrant activation of lipogenesis is a dominant oncogenic event in human HCC. Importantly, no significant differences were detected in the extent of de novo lipogenesis with regard to HCC etiology, suggesting that exacerbated lipogenesis was a general molecular phenotype in hepatocarcinogenesis. Indeed, previous reports have demonstrated that both hepatitis B and C viruses are able to induce FASN expression [47, 48], and over-expression of FASN is a typical feature of liver cancer under another predisposing condition, the alcoholic steatohepatitis [49]. Furthermore, a rat model of insulin-induced hepatocarcinogenesis is characterized by strong up-regulation of FASN [50], which resembles the occurrence of HCC in human affected by type II diabetes mellitus and/or metabolic syndrome, two clinical conditions associated with an increased risk of liver cancer development [51]. Our data suggested that abnormal lipid metabolism was present not only in cancer cells but also in stromal cells. CAFs also expressed a high level of FASN with its maintenance protein USP2a and phosphorylated ACLY. Abnormal lipid accumulation was also coupled with the activation of HSCs.

## Conclusions

Our results indicated that tumor-derived exosomal miRNA-21 could convert HSCs to CAFs by decreasing PTEN, leading to activated PDK1/AKT signaling pathway in HCC. In addition, CAFs exhibited increased secretion of VEGF, MMP2, MMP9, bFGF and TGF-β, promoting angiogenesis. Meanwhile, we found aberrant activation of lipogenesis in activated HSCs. More importantly, high expression of miRNA-21 in serum exosomes showed a positive correlation with survival in HCC patients. Our study elucidated a new molecular mechanism underlying the crosstalk between tumor cells and HSCs during tumor progression, which greatly contributed to efficient prevention and therapeutic strategies for liver cancer.

## Additional files

Additional file 1: **Table S1.** Sequences of primers and miRNA-inhibitor/mimic used in the study. (DOCX 14 kb)

Additional file 2: **Figure S1.** The expression of α-SMA in normal liver tissue was negative. (TIF 740 kb)

Additional file 3: **Figure S2.** Tumor-derived exosomes activated HSCs in vitro. a Flow cytometry assays of cell cycle showed the increasing S phase in HCC derived-exosomes treated HSCs. b HSCs exhibited stronger cell contractility during the stimulation of HCC derived-exosomes. c Proinflammatory cytokines of activated HSCs were identified to be upregulated by HCC derived-exosomes treatment. (TIF 1541 kb)

Additional file 4: **Figure S3.** Tumor-derived exosomes activated HSCs in vivo. a Immunofluorescence imaging showed the delivery of 97H-labeled exosomes (green) to FAP-labeled CAFs (red). Yellow arrows represent delivered exosomes. (TIF 192 kb)

Additional file 5: **Figure S4.** Tumor-derived exosomes activated HSCs in vivo. a Immunohistochemistry imaging of Ki67 showed the proliferation of HSCs with the stimulation of HCC derived exosomes. Black arrows show proliferated cells, white arrows indicate non-proliferated cells. (TIF 713 kb)

Additional file 6: **Figure S5.** Detection of miRNA-21 in HCC cells and HCC cell-derived exosomes treated HSCs. qPCR array demonstrated the high expression of miRNA-21 in HCC cell lines and increased expression of HSCs treated with HCC cell-derived exosomes. (TIF 1120 kb)

Additional file 7: **Figure S6.** MiRNA-21 mediates HSC activation. Cell contraction assay (a), Edu staining assay (b) and flow cytometry assay of cell cycle (c) were used to detect the activation of HSCs transfected with miR-21 mimic or negative control (miR-RC). (TIF 1626 kb)

Additional file 8: **Figure S7.** Exosomal miRNA-21 activates HSCs via PTEN/PDK1/AKT signaling axis. Immunofluorescence assay of α-SMA (a), Edu staining assay (b, c), flow cytometry assay (d), migration assay (e, f), wound-healing assay (g) of HSCs treated with exosomes derived from different cells co-cultured with miRNA-21 inhibitor or AKT inhibitor. Representative images were shown, and migrated cells were counted. (TIF 1699 kb)

Additional file 9: **Figure S8.** Exosomal miRNA-21 activates HSCs via PTEN/PDK1/AKT signaling axis. The HSCs were treated with exosomes derived from different cells co-cultured with miRNA-21 inhibitor or AKT inhibitor. And the cell contraction assay (a), CCK-8 proliferation assay (b) were used to detect the activation of HSCs. c qPCR array demonstrated that the downregulation of proinflammatory cytokines was caused by inhibition of miRNA-21 and AKT activation. (TIF 1736 kb)

Additional file 10: **Figure S9.** Activated HSCs promote angiogenesis. a Immunofluorescence imaging showed the activated CAFs (FAP) and the vessels (red). Yellow arrows represent activated CAFs. (TIF 415 kb)

## Abbreviations

ACLY: ATP citrate lyase
AKT: v-akt murine thymoma viral oncogene homolog
bFGF: Basic fibroblast growth factor
FAP: Fibroblast activation protein
FASN: Fatty acid synthase
FSP: Fibroblast stimulated protein
HCC: Hepatocellular carcinoma
MMP2: Matrix metalloproteinase 2
MMP9: Matrix metalloproteinase 9
PDK1: 3-Phosphoinositide-dependent protein kinase-1
PTEN: Gene of phosphate and tension homology deleted on chromsome ten
TGF-β: Tumor Growth Factor-β
USP2: Ubiquitin-specific protease 2
VEGF-α: Vascular endothelial growth factor-α
α-SMA: α-smooth muscle actin
