# Correction: Hepatocellular carcinoma-derived exosomal miRNA-21 contributes to tumor progression by converting hepatocyte stellate cells tocancer-associated fibroblasts

**DOI:** 10.1186/s13046-022-02575-z

**Published:** 2022-12-27

**Authors:** Yuan Zhou, Haozhen Ren, Bo Dai, Jun Li, Longcheng Shang, Jianfei Huang, Xiaolei Shi

**Affiliations:** 1 Department of Hepatobiliary Surgery, Affiliated Drum Tower Hospital of Nanjing University Medical School, 321, Zhongshan Road, Nanjing, 210008 Jiangsu Province China; 2 Department of Clinical Biobank, Nantong University Affiliated Hospital, 20, Xisi Road, Nantong, 226001 Jiangsu Province China

**Correction:** *J Exp Clin Cancer Res* **37, 324 (2018)**

**https://doi.org/10.1186/s13046-018-0965-2**

Following the publication of the original article [1], authors identified errors in Figs. 3, 5 and 6, specifically:

- Figure 3c and 3f – 1 cm scale mark missing
- Figure 5e - P-PTEN stripe was repeated in Figure 5c PDK1 stripe
- Figure 6b - UPS2a stripe were repeated

**Fig. 3:**
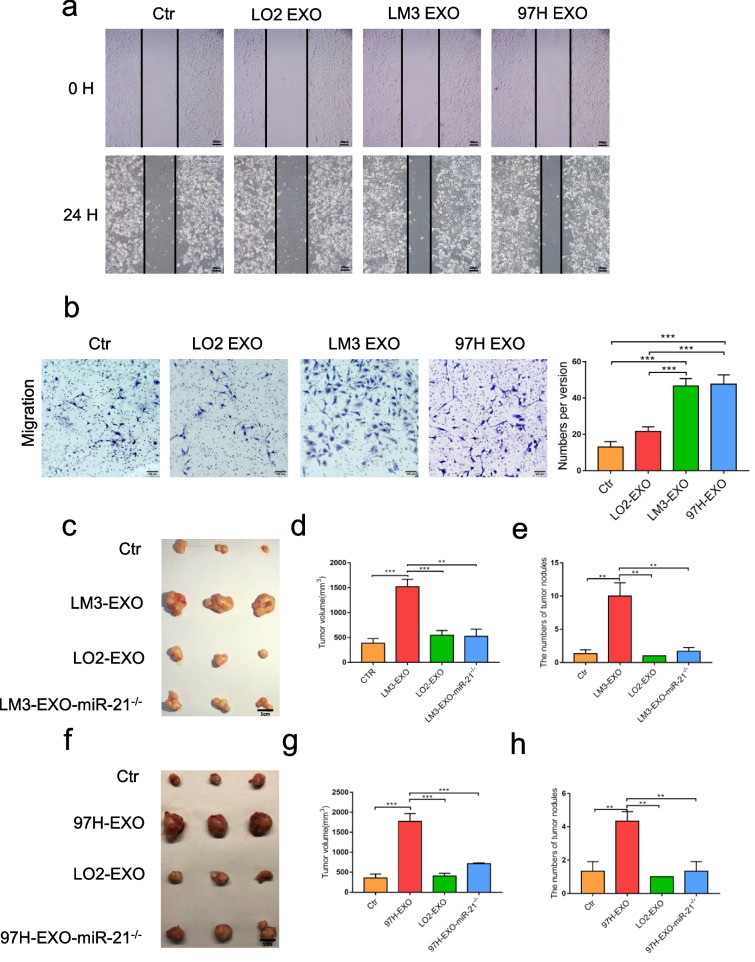
Tumor-derived exosomes activated HSCs in vitro. Wound-healing assays (**a**) and migration assay (**b**) of HSCs treated with equal quantities of exosomes derived from different liver cancer cells or blank control. **c** – **h** Xenograft assays of Huh7 with indicated treatments were performed on nude mice. Representative tumors, tumor volume and number of tumor nudes were shown. Experiments were performed at least in triplicate, and results are shown as mean ± s.d. Student’s t-test was used to analyze the data (NS, not significant; *p < 0.05; **p < 0.01; ***p < 0.001)

**Fig. 5:**
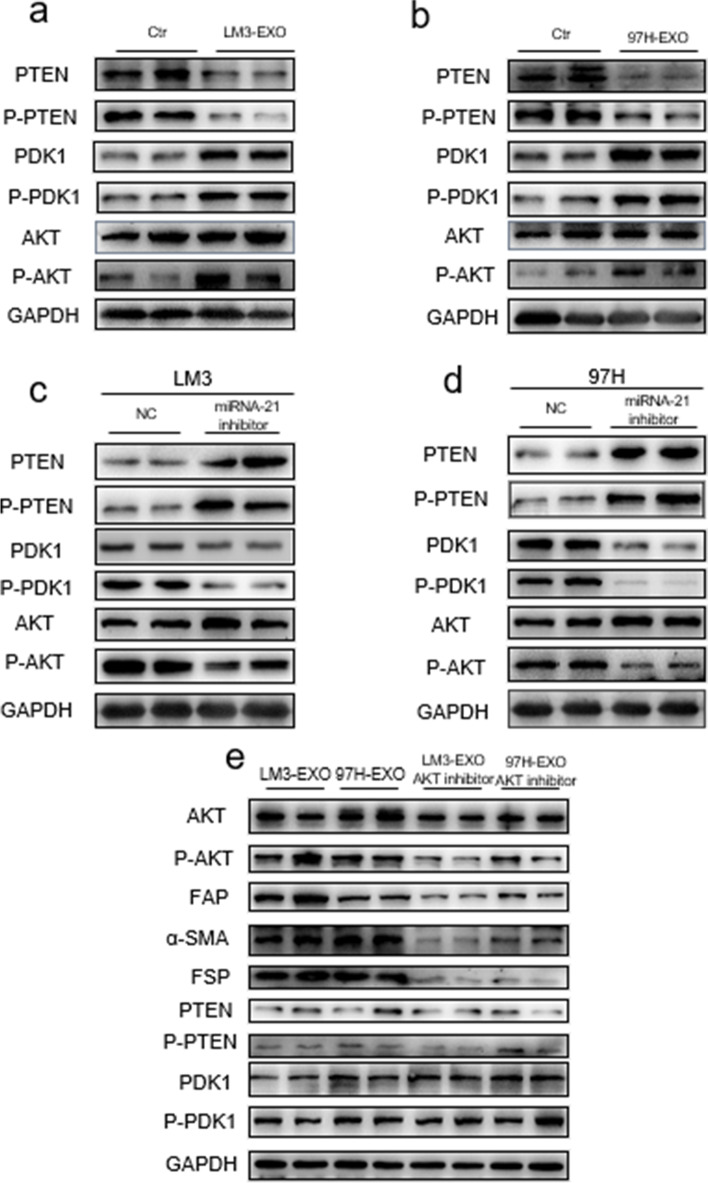
Exosomal miRNA-21 activates HSCs via PTEN/PDK1/AKT signaling axis. **a**,** b** Immunoblotting assays of indicated proteins in HSCs treated with control or exosomes from different tumor cells. **c-e** Western blotting assays of indicated proteins in HSCs with indicated treatments. Each experiment was performed in triplicate, and data are presented as mean ± s.d. Student’s t-test was used to analyze the data (*p < 0.05; **p < 0.01; ***p < 0.001)

**Fig. 6:**
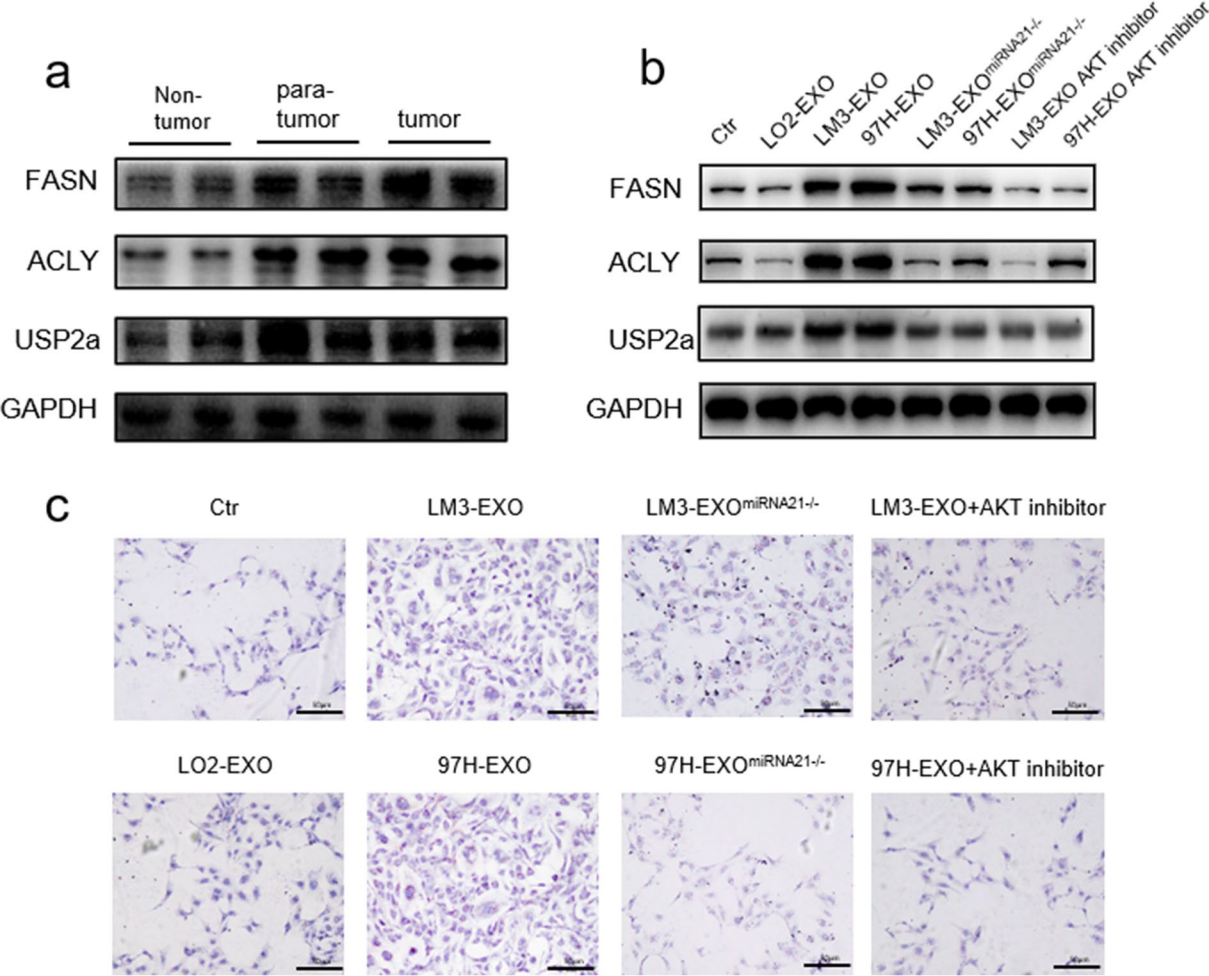
HCC derived exosomes induced abnormal lipid metabolism. **a**,** b** Western blotting assays of lipid metabolism related proteins in HCC patients or HSCs with different stimulations. **c** Oil Red staining assay showed the abnormal lipid accumulation in HSCs with indicated treatments. Each experiment was performed in triplicate, and data are presented as mean ± s.d. Student’s t-test was used to analyze the data (*p < 0.05; **p < 0.01; ***p < 0.001)

The corrected figures are also provided below.
