# Supplementary material for: Hepatocellular carcinoma-derived exosomal miRNA-21 contributes to tumor progression by converting hepatocyte stellate cells to cancer-associated fibroblasts
Source: J Exp Clin Cancer Res. 2018 Dec 27;37:324. doi: 10.1186/s13046-018-0965-2 (PMC6307162; doi:10.1186/s13046-018-0965-2)
Supplement: Supplementary file 1 — Table S1. Sequences of primers and miRNA-inhibitor/mimic used in the study. (DOCX 14 kb) [file 13046_2018_965_MOESM1_ESM.docx]

**Additional file 1: Table 1**

| Gene | Sequences |
| --- | --- |
| MiRNA-21 | 5′- TTTTGTTTTTGCTGGTCTTAG -3’  5’- AGCAGACAGTCAGGCAGGAT -3’ |
| MiRNA-27a | 5’- GCGGCGGTTCACAGTGGCTAAG -3’  5’- ATCCAGTGCAGGGTCCGAGG -3’ |
| MiRNA-34 | 5’- ACAGUAGUCUGCACAUUGGUUA -3’  5’- UGGCAGUGUCUUAGCUGGUUGU -3’ |
| MiRNA-122 | 5’- GACGGCGCTAGGATCATCAAC -3’  5’- ATTCTGTGACCAGAATAC -3’ |
| MiRNA-126 | 5’- CATTATTACAGGGCAGCGGTCGC -3’  5’- CATTATTACG CGGCAGGTGCCGT -3’ |
| U6 | 5’- GCTTCGGCAGCACATATACTAAAAT -3’  5’- CGCTTCACGAATTTGCGTGTCAT -3’ |
| VEGF-α | 5’- CCCTGGCTTTACTGCTGTAC -3’  5’- TCTGAACAAGGCTCACAGTG -3’ |
| MMP2 | 5’- CTTCTTCCCTCGCAAGCC -3’  5’- ATGGATTCGAGAAAACCG -3’ |
| MMP9 | 5’- ACGCAGACATCGTCATCC -3’  5’- AACCGAGTTGAACCACG -3’ |
| bFGF | 5’- TGGCTTCTAAATGTGTTACG -3’  3’- GTTTATACTGCCCAGTTCG -3’ |
| GAPDH | 5’- AACGGATTTGGTCGTATTGGG -3’  5’- CCTGGAAGATGGTGATGGGAT -3’ |
| TGFβ | 5’- AAACTAAGGCTCGCCAGTCC -3’  5΄- TTGTTGCGGTCCACCATT -3’ |
| IL-8 | 5’ -TGCAGCTCTGTGTGAAGGTG -3’  5’ - ACTTCTCCACAACCCTCTGC -3’ |
| IL-6 | 5’ -CCTTCCAAAGATGGCTGAAA-3’  5’ -AGCTCTGGCTTGTTCCTCAC-3’ |
| IL-1 | 5’ -GCATCCAGCTACGAATCTCC-3’  5’ -TCGTTATCCCATGTGTCGAA -3’ |
| SCD1 | 5’ -GGGCAAAGCCTAGTGAAGG-3’  5’ -ATGAGCAGAACGTGGAGGAT-3’ |
| CCL5 | 5’ -CGCTGTCATCCTCATTGCTA-3’  5’ -CCAGACTTGCTGTCCCTCTC-3’ |
| CCL2 | 5’ -TCTGTGCCTGCTGCTCATAG-3’  5’ -CATGGAATCCTGAACCCACT-3’ |
| MiRNA-21 inhibitor | 5’- UAGCUUAUCAGACUGAUGUUGA-3’  5’-AUCGAAUAGUCUGACUACAACU-3’ |
| MiRNA-21 mimic | 5’-AUCGAAUAGUCUGACUACAACU-3’ |
|  |  |
