# Supplementary figures and images for: Hepatocellular carcinoma-derived exosomal miRNA-21 contributes to tumor progression by converting hepatocyte stellate cells to cancer-associated fibroblasts
Source: J Exp Clin Cancer Res. 2018 Dec 27;37:324. doi: 10.1186/s13046-018-0965-2 (PMC6307162; doi:10.1186/s13046-018-0965-2)

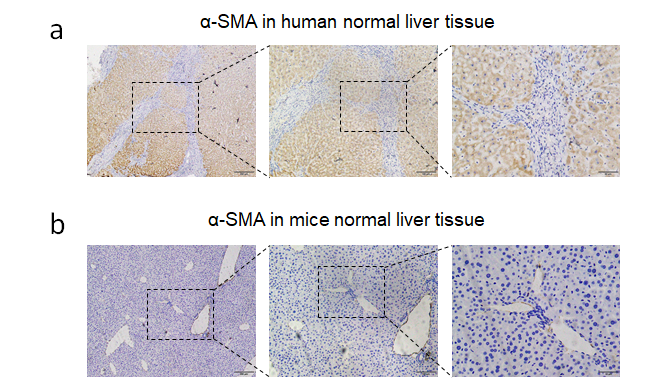

Supplement: Supplementary file 2 — Figure S1. The expression of α-SMA in normal liver tissue was negative. (TIF 740 kb) [file 13046_2018_965_MOESM2_ESM.tif]

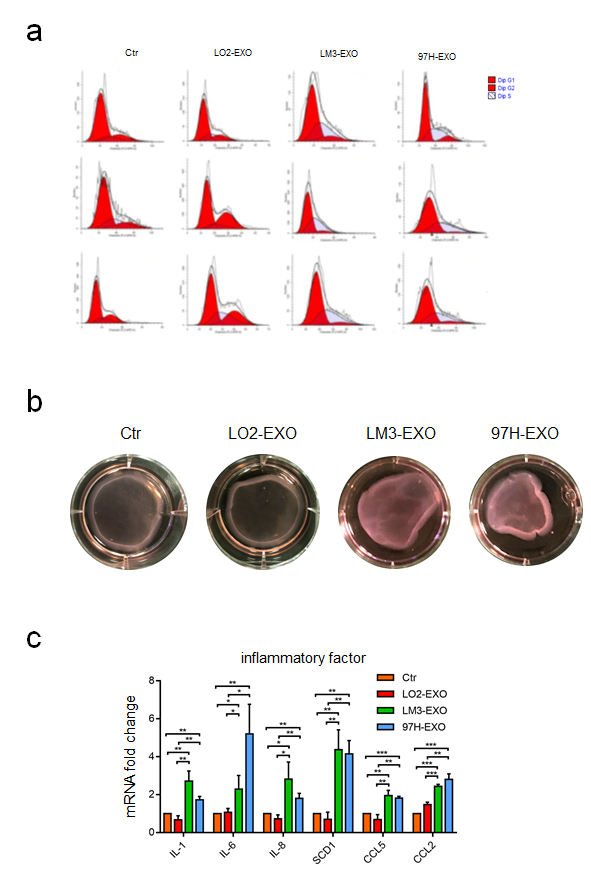

Supplement: Supplementary file 3 — Figure S2. Tumor-derived exosomes activated HSCs in vitro. a Flow cytometry assays of cell cycle showed the increasing S phase in HCC derived-exosomes treated HSCs. b HSCs exhibited stronger cell contractility during the stimulation of HCC derived-exosomes. c Proinflammatory cytokines of activated HSCs were identified to be upregulated by HCC derived-exosomes treatment. (TIF 1541 kb) [file 13046_2018_965_MOESM3_ESM.tif]

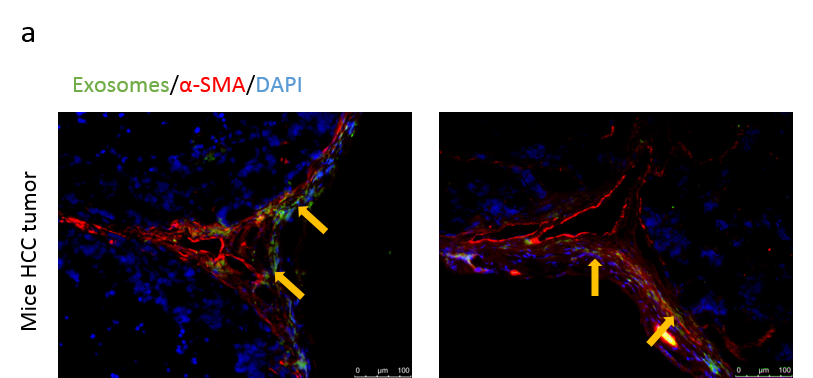

Supplement: Supplementary file 4 — Figure S3. Tumor-derived exosomes activated HSCs in vivo. a Immunofluorescence imaging showed the delivery of 97H-labeled exosomes (green) to FAP-labeled CAFs (red). Yellow arrows represent delivered exosomes. (TIF 192 kb) [file 13046_2018_965_MOESM4_ESM.tif]

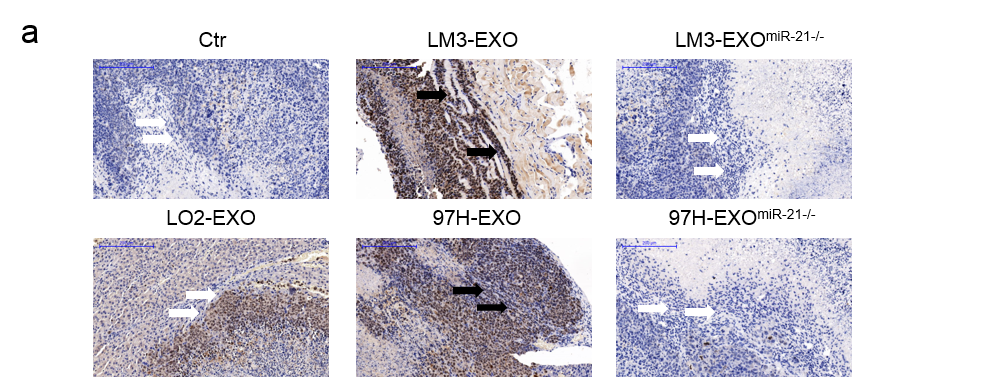

Supplement: Supplementary file 5 — Figure S4. Tumor-derived exosomes activated HSCs in vivo. a Immunohistochemistry imaging of Ki67 showed the proliferation of HSCs with the stimulation of HCC derived exosomes. Black arrows show proliferated cells, white arrows indicate non-proliferated cells. (TIF 713 kb) [file 13046_2018_965_MOESM5_ESM.tif]

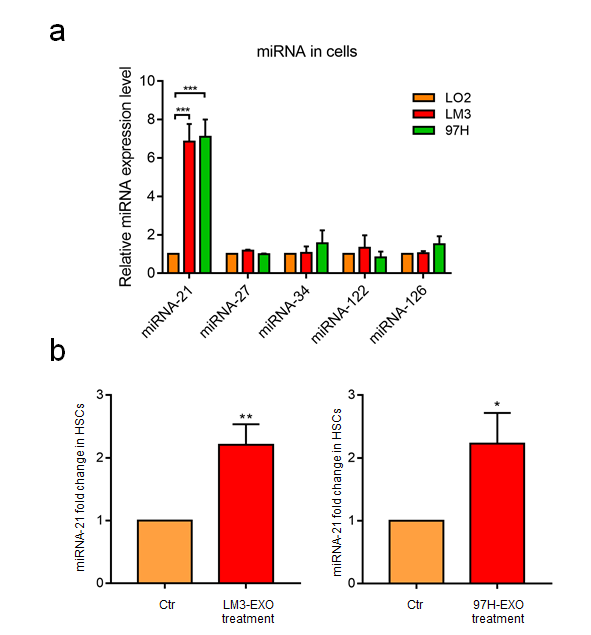

Supplement: Supplementary file 6 — Figure S5. Detection of miRNA-21 in HCC cells and HCC cell-derived exosomes treated HSCs. qPCR array demonstrated the high expression of miRNA-21 in HCC cell lines and increased expression of HSCs treated with HCC cell-derived exosomes. (TIF 1120 kb) [file 13046_2018_965_MOESM6_ESM.tif]

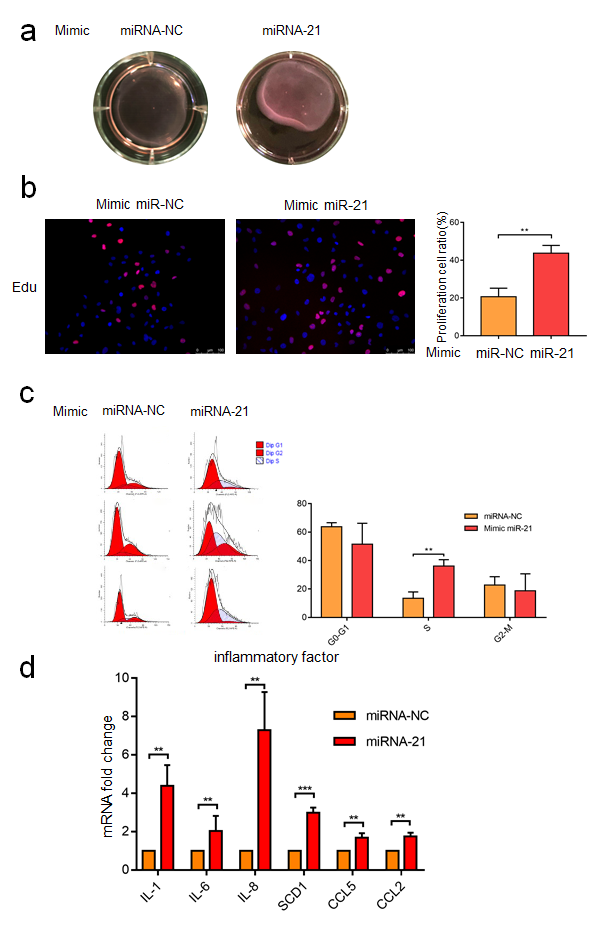

Supplement: Supplementary file 7 — Figure S6. MiRNA-21 mediates HSC activation. Cell contraction assay (a), Edu staining assay (b) and flow cytometry assay of cell cycle (c) were used to detect the activation of HSCs transfected with miR-21 mimic or negative control (miR-RC). (TIF 1626 kb) [file 13046_2018_965_MOESM7_ESM.tif]

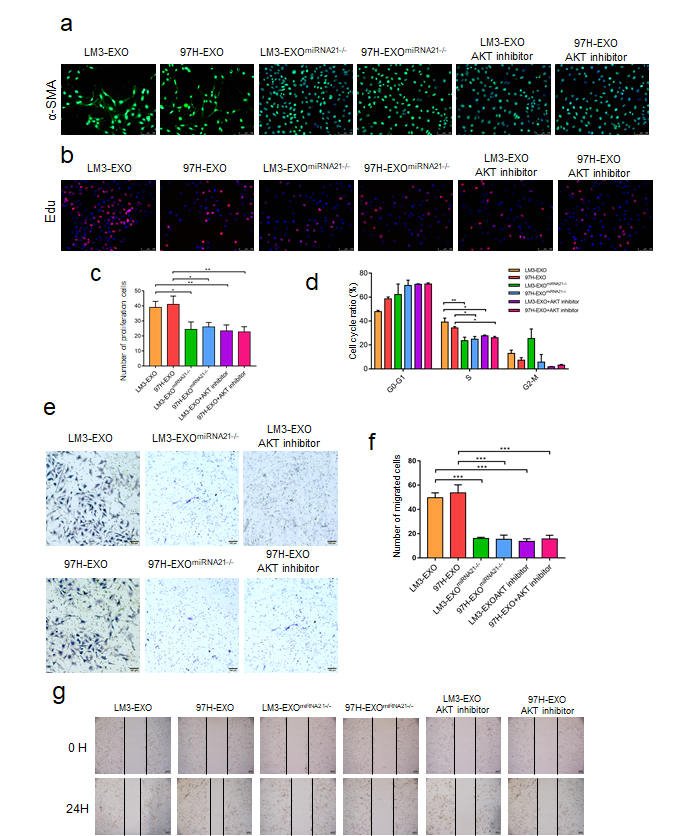

Supplement: Supplementary file 8 — Figure S7. Exosomal miRNA-21 activates HSCs via PTEN/PDK1/AKT signaling axis. Immunofluorescence assay of α-SMA (a), Edu staining assay (b, c), flow cytometry assay (d), migration assay (e, f), wound-healing assay (g) of HSCs treated with exosomes derived from different cells co-cultured with miRNA-21 inhibitor or AKT inhibitor. Representative images were shown, and migrated cells were counted. (TIF 1699 kb) [file 13046_2018_965_MOESM8_ESM.tif]

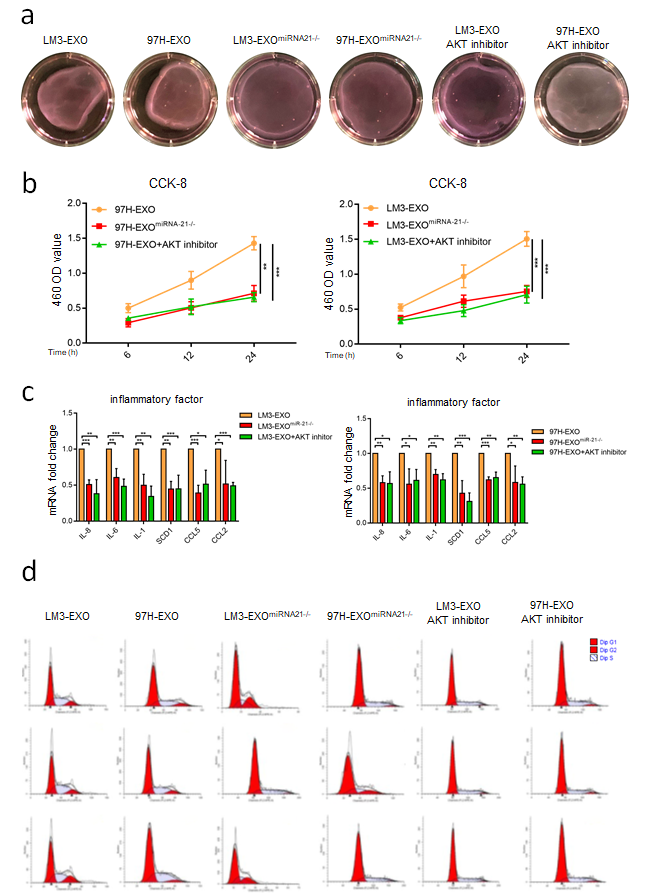

Supplement: Supplementary file 9 — Figure S8. Exosomal miRNA-21 activates HSCs via PTEN/PDK1/AKT signaling axis. The HSCs were treated with exosomes derived from different cells co-cultured with miRNA-21 inhibitor or AKT inhibitor. And the cell contraction assay (a), CCK-8 proliferation assay (b) were used to detect the activation of HSCs. c qPCR array demonstrated that the downregulation of proinflammatory cytokines was caused by inhibition of miRNA-21 and AKT activation. (TIF 1736 kb) [file 13046_2018_965_MOESM9_ESM.tif]

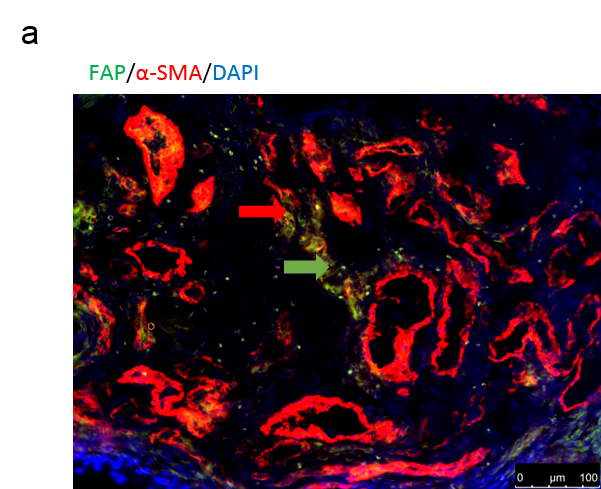

Supplement: Supplementary file 10 — Figure S9. Activated HSCs promote angiogenesis. a Immunofluorescence imaging showed the activated CAFs (FAP) and the vessels (red). Yellow arrows represent activated CAFs. (TIF 415 kb) [file 13046_2018_965_MOESM10_ESM.tif]
